# Polymer-Based Hydrogels Enriched with Essential Oils: A Promising Approach for the Treatment of Infected Wounds

**DOI:** 10.3390/polym14183772

**Published:** 2022-09-09

**Authors:** Sibusiso Alven, Sijongesonke Peter, Blessing Atim Aderibigbe

**Affiliations:** Department of Chemistry, Faculty of Science and Agriculture, Alice Campus, University of Fort Hare, Alice 5700, Eastern Cape, South Africa

**Keywords:** wound dressing, polymers, hydrogels, essential oils, lavender oil, infected wounds, tea tree oil

## Abstract

Among the factors that delay the wound healing process in chronic wounds, bacterial infections are a common cause of acute wounds becoming chronic. Various therapeutic agents, such as antibiotics, metallic nanoparticles, and essential oils have been employed to treat infected wounds and also prevent the wounds from bacterial invasion. Essential oils are promising therapeutic agents with excellent wound healing, anti-inflammatory and antimicrobial activities, and good soothing effects. Some essential oils become chemically unstable when exposed to light, heat, oxygen, and moisture. The stability and biological activity of essential oil can be preserved via loading into hydrogels. The polymer-based hydrogels loaded with bioactive agents are regarded as ideal wound dressings with unique features, such as controlled and sustained drug release mechanisms, good antibacterial activity, non-toxicity, excellent cytocompatibility, good porosity, moderate water vapour transmission rate, etc. This review addresses the pre-clinical outcomes of hydrogels loaded with essential oils in the treatment of infected wounds.

## 1. Introduction

Chronic wounds remain a huge challenge in the biomedical fields due to their slow healing process and some do not heal at all. Several factors make wounds chronic, including bacterial infections, underlying health conditions (such as diabetes and cancer), malnutrition, obesity, and smoking [1,2]. Statistics have shown that chronic and acute wounds affect over 2% of the world population, with treatment costs taking up to 4% of the overall health care budget [3]. The cost of managing a patient that suffers from chronic injury can cost up to 6000–10,000 EUR per annum in Europe [4]. Among the approaches that are used for the management of wounds, wound dressings that are formulated from polymers (synthetic polymers and biopolymers) are potential materials, although they also suffer from some limitations. Limitations associated with some of the currently used polymer-based wound dressing materials include their poor absorption of wound exudates; inability to maintain a moist environment; poor mechanical properties, retarded process of wound recovery (i.e., epidermal migration, connective tissue synthesis, and angiogenesis); poor antibacterial activity; difficulty in removal of the wound dressing after healing; and their capability to cause allergic reactions [5]. These shortcomings are commonly overcome by combining biopolymers and synthetic polymers to form hybrid-based wound dressings with improved properties. Wound dressings are generally classified into different categories: skin substitutes, dermal grafts, traditional dressings, interactive dressings, and bioactive dressings [6]. 

Skin substitutes and dermal grafts are used to replace disrupted skin [7]. Dermal grafts include autografts, acellular xenografts, and allografts [8]. The limitations of skin substitutes and dermal grafts are short survival time on wound site, host rejection, and possibilities of disease transmission [7,8]. Traditional dressings are mainly used to keep the wound safe from contamination or foreign substances, cushion the wound, and absorb wound exudate, and examples include wool dressing, plaster, gauze, and bandages [9]. The limitation of traditional dressings is the possibility of skin re-damage and pain if they are not changed frequently [9]. Interactive dressings are employed as the barrier against bacterial infections, offering a moist environment to accelerate the wound healing process, displaying good water transmission, and enhancing re-epithelialization and granulation, and examples include films, gels, and foams. The shortcoming of interactive dressings is their limited antibacterial efficacy [10]. Bioactive dressings are biocompatible and biodegradable and are mainly used as drug delivery systems for various bioactive agents, such as antibiotics, metal-based nanoparticles, growth factors, and essential oils to accelerate the wound healing process [11]. Examples of bioactive wound dressings include hydrogels, nanofibers, sponges, and transdermal patches. Hydrogels are characterized by a cross-linked polymeric network that can retain a significant amount of water in their networks. Their elasticity is similar to the native tissue, provides a moist environment to the wound bed, absorbs wound exudates due to its porous network, promotes a good gaseous exchange useful in inhibiting bacterial growth, induces epithelization and cell migration and supports tissue regeneration, making them useful for wound healing applications [12]. 

**Figure 1:**
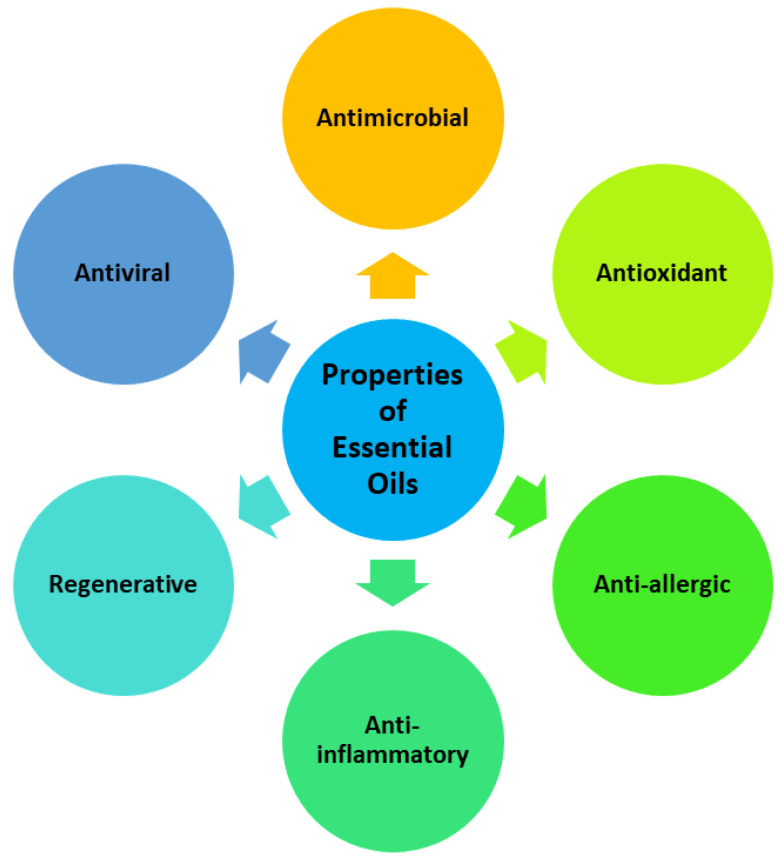
Biomedical properties of essential oils.

Essential oils are also known as volatile natural mixtures that exhibit anti-inflammatory, antiviral, antimicrobial, antioxidant, anti-allergic, and regenerative properties (Figure 1) [13]. These oils are commonly extracted from vegetable parts of plants (such as roots, twigs, barks, seeds, and leaves). The extraction methods that are used for the synthesis of essential oils include hydrodistillation, microwave-assisted extraction, steam distillation, microwave-generated hydrodistillation, microwave steam diffusion, and ultrasound-assisted extraction. Essential oils are normally used in first aid treatment of wounds, abscesses, or burns. Researchers demonstrated that the antimicrobial activity, antioxidant, and wound-healing promoting properties of essential oils enriched in wound dressings could be ascribed to their various constituents (such as geraniol, cinnamaldehyde, eucalyptol, thymol, carvacrol, menthol, etc.) [14]. This review is focused on pre-clinical experiments and outcomes of polymer-based hydrogels enriched with essential oils for the treatment of wounds. 

## 2. Phases of Wound Healing Process

**Figure 2:**
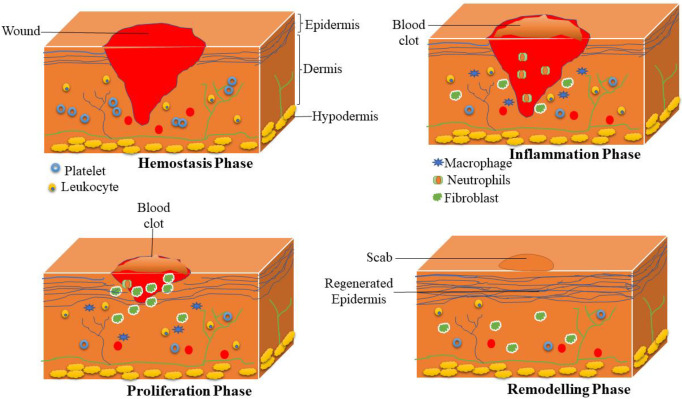
Sequential phases of the wound healing process.

Wound healing is defined as a complex process that is commonly divided into four sequential and sometimes overlapping phases: hemostasis, inflammation, proliferation (tissue growth), and maturation phase (tissue remodelling) (Figure 2) [15]. In the hemostasis phase (blood clotting), blood platelets begin to attach to the wound bed immediately after the wound. Platelets turn into an amorphous shape when they are in contact with collagen, leading to their aggregation. Thrombin is produced and induces the initiation of the coagulation cascade, leading to the activation of fibrin that produces mesh, which stops further bleeding [16,17]. The macrophages and neutrophils are recruited into the injury during the inflammatory phase, where the macrophages engulf dead cells, bacteria, and debris. Furthermore, inflammatory cells release various growth factors (e.g., endothelial growth factors, fibroblast growth factor, etc.) that stimulate the invasion of fibroblast into the wound bed and trigger angiogenesis [18]. 

In the proliferation phase, the fibroblasts are further induced to proliferate in the wound site. Moreover, they reconstruct the components of dermal tissue by the development of granulation tissue and deposition of extracellular matrix proteins, particularly collagen [19]. In addition, matured angiogenesis stimulates the ingrowth of a new network of blood vessels inside the granulation tissue to improve the survival of the cells by offering enough levels of nutrients and oxygen. Then, the process called epithelialization occurs whereby epithelial cells migrate from the injury edges to cover the defect [20]. The final phase of the wound healing process is called remodelling, also named the maturation phase; the numerous fibres of collagen are degraded in the skin with skin contraction. The healing wound tissue reaches its maximum tensile strength. The final resultant scar will have 80% of the original strength of the injury [21].

## 3. Classification and Properties of Polymers in Wound Healing Applications

**Figure 3:**
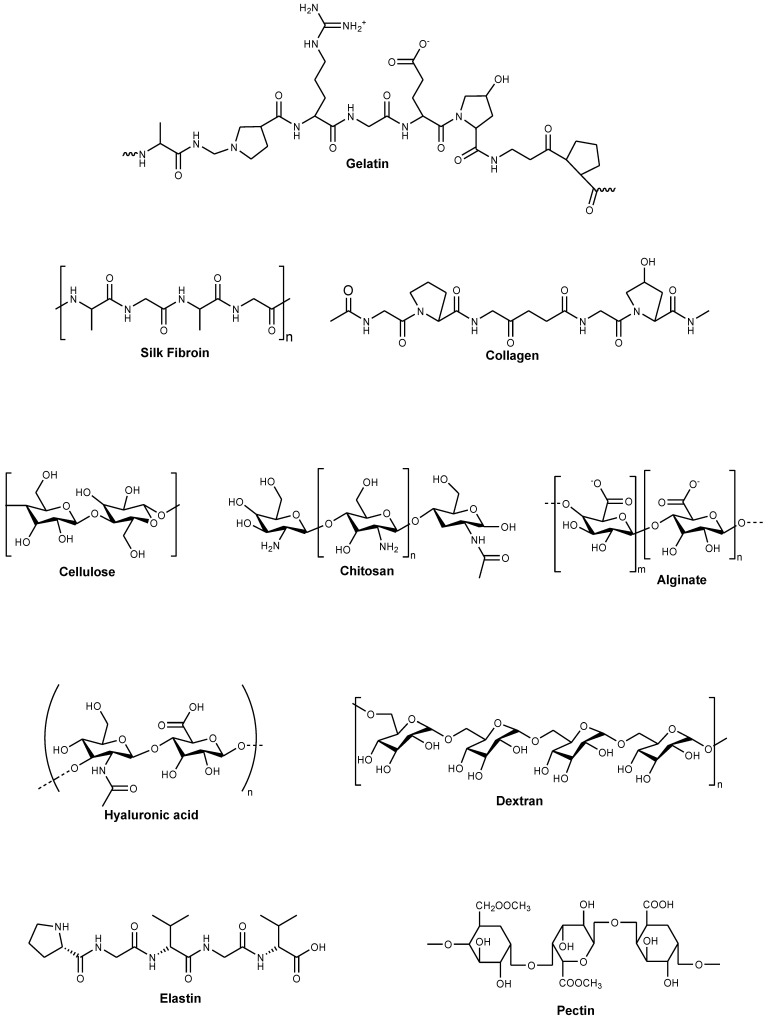
Molecular structures of some natural polymers.

Polymers are defined as materials that consist of very large molecules made up of numerous repeating subunits. A broad variety of polymer-based scaffolds that can be used in wound dressings have attracted remarkable attention within the healthcare community for the treatment of chronic wounds. Polymers are usually classified into two groups: synthetic and biopolymers. Biopolymers are macromolecules that are derived from animal, plant, and microbial sources [22]. Although biopolymers can nearly mimic the original extracellular matrix (ECM) and cellular milieus, they are importantly heterogeneous and may undergo significant biodegradation that can alter their physicochemical features and their behaviour (e.g., interaction with surrounding cells, release mechanism, etc.) in the biological environment [23]. Examples of natural polymers include chitosan, cellulose, alginate, hyaluronic acid (HA), gelatin, collagen, elastin, pectin, etc. (Figure 3) [24,25]. Some properties of natural polymers make them useful in wound dressing applications, such as their non-toxicity, antigenicity, good biocompatibility and biodegradability, good mucoadhesive properties, the drug delivery ability of various bioactive agents, strong attachment to wound tissues, and capability to stimulate blood clotting and wound healing [26]. Nevertheless, the common limitation of natural polymers is their poor mechanical performance, which can be minimized by hybridizing them with synthetic polymers [27].

**Figure 4:**
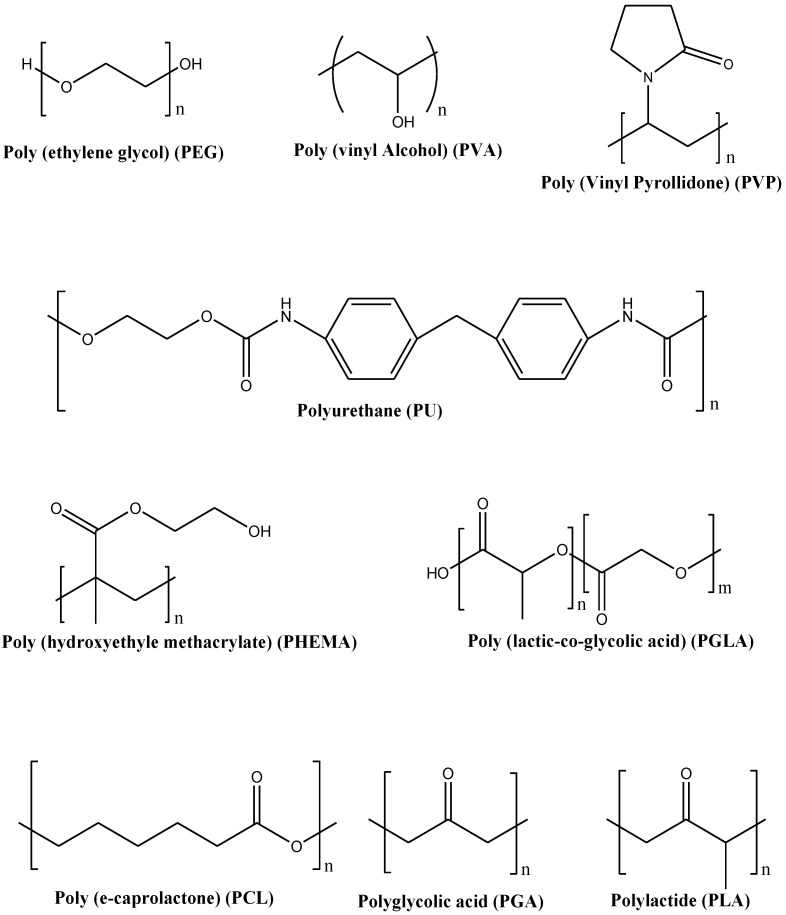
Molecular structures of some synthetic polymers.

A wide variety of synthetic polymers and copolymers that are biodegradable, non-biodegradable and biocompatible have been developed in recent decades. These polymers overcome the shortcomings of biopolymers by achieving reproducible and stable physical and chemical properties [28]. Additionally, most synthetic polymers are insensitive to enzymatic and biological activities, and hence their biochemical and physicochemical performance will not vary significantly from patient to patient [29]. Wound dressings that are formulated from synthetic polymers exhibit excellent mechanical properties, making them easily used during wound dressing application. Examples of synthetic polymers include Poly (ethylene glycol) (PEG)/Poly (ethylene oxide) (PEO), Poly (vinyl alcohol) (PVA), Poly (vinyl pyrrolidone) (PVP), Polyurethanes (PUs), Poly (hydroxyethyl methacrylate) (PHEMA), Polylactide (PLA), Polyglycolic acid (PGA), Poly (lactic-co-glycolic acid) (PLGA), and Polycaprolactone (PCL) (Figure 4) [30,31,32,33]. Some of the interesting properties of synthetic polymers include their hydrophilic nature, non-toxicity, non-immunogenicity, non-carcinogenicity, tissue regeneration and wound healing properties, strong water absorption and oxygen permeability properties [28].

## 4. Different Forms of Hydrogels and Some Reported Progress in the Design of Hydrogels

**Figure 5:**
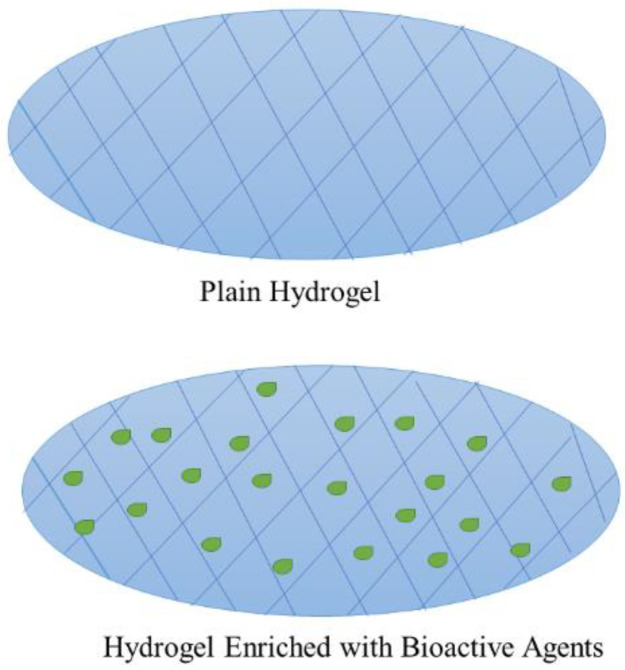
Plain and hydrogel loaded with bioactive agents.

Hydrogels are three-dimensional network scaffolds that are composed of hydrophilic polymers with excellent swelling and absorption capacity of water and other biological fluids without dissolving (Figure 5) [34]. Hydrogels consist of around 90% water and 10% synthetic or natural polymers; this high water content makes them wound dressings appropriate for the treatment of necrotic and dry injuries [35]. These scaffolds have been widely employed in biomedical and pharmaceutical applications because of their ability to accurately mimic the natural ECM properties. Consequently, in wound dressing applications, hydrogels not only provide a physical barrier with a good absorption capacity of excess wound exudate but can also be loaded with bioactive agents and offer a moist environment that stimulates the process of wound healing [36,37]. Furthermore, injectable hydrogels can completely cover irregular-shaped injuries and deep bleeding wounds more effectively [38]. Due to the many benefits of hydrogels, a series of hydrogels that are commercially available for wound treatment have been developed, such as Coseal^®^, Evicel^®^, Tegaderm^TM^ hydrocolloid dressing, and Algisite M [39]. The novel hydrogel materials with various properties (e.g., biodegradability, antibacterial ability, promote wound healing, high porosity, injectability, and responsiveness) have attracted much attention in the field of wound healing in recent years due to the demand for higher performance wound dressings [36,39,40,41]. To improve some of the properties of hydrogel wound dressings, such as antibacterial or antioxidant activity, they can be enriched with bioactive agents that include essential oils. The hydrogel loaded with bioactive agents is shown in Figure 5 below. Hydrogel materials are normally categorized into three groups: in situ forming hydrogels, acellular hydrogels, and hydrogels with integrated sensors.

In situ forming hydrogels that are called “smart” wound dressing materials are potential materials that overcome shortcomings associated with conventional dressing products that are utilized in clinical applications. These materials can be simply crosslinked by employing various physical or chemical methods such as ionic-, photo-, or thermal crosslinking [42,43]. Sprayable hydrogel dressings are among the different kinds of “smart” hydrogels that are appropriate “in situ” forming dressings to treat lesions. These hydrogels display many advantages, such as low production costs, improved patient compliance, simple application without the help of a specialist, reduced frequency of administration, and extended contact time of drug at the site (thereby enhancing drug bioavailability) [44]. Furthermore, spray delivery can increase the diffusion of the nanocomposite hydrogel into the wound site, thereby contributing to the enhanced delivery of bioactive agents to the injury. Nevertheless, the preparation of sprayable hydrogels needs to be cautiously adjusted to have an ideal viscosity to be applied as a spray-on dressing and uniformly cover the wound environment [45]. 

On the other hand, acellular hydrogels are hydrogel materials that are produced in the form of sheets or films. These materials can be fabricated from biopolymers (natural polymers) or synthetic crosslinked polymers (e.g., poly (poly-vinylpyrrolidone, methacrylates), polyurethane, and polyvinyl alcohol) [46]. There are different types of hydrogel scaffolds based either on synthetic polymers or natural components. Biopolymer hydrogels are regularly employed as dermal skin substitutes because of their unique features such as flexibility, softness, biocompatibility, and high-water content [47]. Synthetic polymer hydrogels utilized as skin templates represent several benefits when compared with naturally derived polymers. They exhibit controllable and predictable characteristics, such as low production costs, easy shape control, and stable mechanical properties [48]. Moreover, synthetic hydrogels are convenient to prepare as stable formulations. However, synthetic hydrogel materials should be chosen precisely to prevent the risk of transplant rejection and disease transmission [48]. Lastly, hydrogels with integrated sensors are hydrogel materials that can offer significant information about the conditions of the wound, such as its pH, temperature, bacterial density, inflammation level, and degree of oxygenation [49]. Recently, different sensors have been developed to measure various biomarkers, such as temperature, pH, oxygen level, moisture, electrical and mechanical properties of wound or skin, and downregulation or upregulation of enzyme levels [50]. All the sensors should possess some vital characteristics, including proportional flexibility to the hydrogel film and to body contours, resistance to wound exudate, non-toxicity, and biocompatibility. Moreover, for biodegradable wound dressing materials, the integrated sensors should be proportionally degraded with a degradation rate to the hydrogel matrix rate and with non-toxic degradation debris to the immune system [51]. Furthermore, it is essentially significant to develop novel dressings with integrated sensors, which can monitor the early status of the injury [52].

The specific wound healing mechanisms whereby a hydrogel material may cause the regeneration of skin cells to represent an interesting arena of research are still not completely understood. A recent research study has tried to show the biological events that happen at the edges of a chronic injury following the application of various wound dressing materials [53]. Remarkably, while porous alginate and collagen scaffolds were shown to hamper re-epithelialization and increase the inflammatory response, alginate hydrogels did not cause similar responses and seemed to be much more biocompatible. However, the specific hydrogel–tissue interactions that may lead to tissue regeneration have not been fully addressed [53,54,55]. Some of the wound dressing scaffolds showed similar properties as hydrogel materials. Porous wound dressings such as sponges and bandages possess the ability to absorb large amounts of wound exudates and other biological fluids, and they can offer a moist environment for the wound due to their high porosity, swelling profile, and biodegradability [56]. Furthermore, nanofibrous scaffolds mimic the ECM, a similar feature of hydrogels, and promote the development of new tissues and the proliferation of epithelial cells in the wound site [57]. Nevertheless, hydrogel wound dressings possess poor mechanical properties when compared to films and membrane wound dressings [57].

## 5. Hydrogels Enriched with Essential Oils for Wound Healing Applications

Essential oils play a vital role in wound healing, especially in microbial-infected wounds. Essential oils (specifically those that contain phenolic compounds, e.g., carvacrol and thymol) possess the potent capability to act in the three phases of the wound healing process. In the inflammation phase, they induce a modulatory effect of oxidative stress, inflammatory cytokines, and antimicrobial activity. They also induce angiogenesis, re-epithelialization, and growth of granulation tissue. In the remodelling phase, they promote the deposition of collagen and modulate the growth of keratinocytes and fibroblasts [58]. Some research studies discussed the mechanism of action of essential oils against various bacterial strains that are common in wound infections, (such as *staphylococcus aureus*, *Escherichia coli*, etc.). Essential oils (specifically those that contain phenolic compounds, e.g., carvacrol and thymol) attack phospholipids and lipids that are present in the bacteria cell wall and plasma membranes, leading to cytoplasmic outflow, disruption of the cellular process (such as protein synthesis and DNA transcription, and ATP biosynthesis), and decreased pH. The most important benefit is that essential oils possess a very small effect on the development of antimicrobial resistance compared to antibiotics. Nevertheless, essential oils are needed in high concentrations or via a repeated application for enhanced therapeutic outcomes. However, the administration of essential oils in high concentrations can cause some side effects in some patients [59,60].

### 5.1. Hydrogels Enriched with Lavender Oil

**Figure 6:**
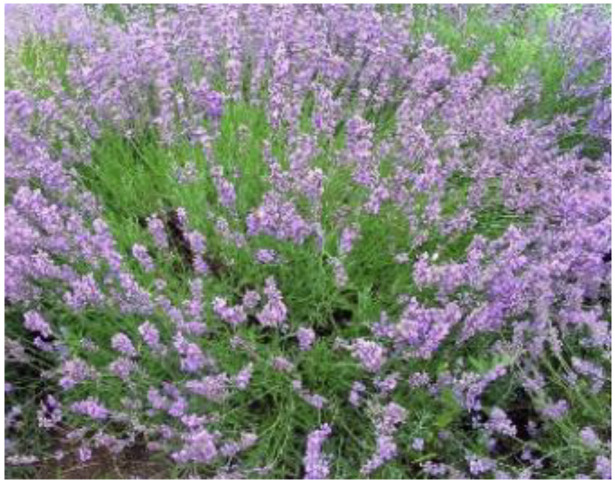
Lavender plant [61].

Lavender oil is an essential oil that originates from many species of the lavender plant. *Lavandula angustifolia*, *Lavandula latifolia*, *Lavandula intermedia* and *Lavandula stoechas* are the most common species used for oil production. A picture of a lavender plant is shown in Figure 6 [61]. This essential oil has been used worldwide in traditional medicine. Lavender essential oil possesses antifungal, antibacterial, carminative, anti-depressive, and sedative properties and is effective for insect bites and burns [62]. Lavender oil effectively hinders the growth of microorganisms that cause infections [63]. The antimicrobial efficacy of lavender oil is due to its major constituents, linalyl, and linalool, but the antibacterial activity and chemical composition of lavender oil is mostly dependent on the sample source of lavender. This essential oil is utilized for the treatment of surface infection in the form of a topical or prophylactic application [62]. Besides the antifungal and antibacterial activity, lavender oil also shows a significant role in enhancing phases of the wound healing process [64]. There are some reported polymer-based hydrogels enriched with lavender oil that demonstrates interesting properties for the treatment of wounds. 

Mahmood et al. formulated gellan gum hydrogels co-enriched with lavender oil and ofloxacin for wound healing application [65]. The Fourier transform infrared (FTIR) and X-ray diffraction (XRD) spectroscope confirmed the successful preparation of hydrogels. Swelling analysis of hydrogels exhibited excellent swelling behaviour followed by an insignificant decrease in the swelling index, which is due to polymeric erosion. The in vitro drug release studies at physiological conditions (pH 7.4, 37 °C) displayed sustained drug release of lavender oil (71%) and ofloxacin (85%) over 48 h; this can help to maintain the drug level for a prolonged period at wound bed. The antioxidant results showed that polymeric hydrogels enriched with lavender oil and ofloxacin possess great potential to scavenge free radicals, which potentiate the wound healing efficacy of hydrogels. Consequently, the in vitro antimicrobial analysis of gellan gum hydrogels co-enriched with lavender oil and ofloxacin exhibited good antibacterial activity against negative (*E. coli*) and Gram-positive (*S. aureus*) bacteria, suggesting that these hydrogels are effective scaffolds that can be used for the treatment of bacteria-infected wounds. In addition, the in vivo wound healing experiments using a full thickness wound model on rats showed an almost complete wound closure of 98% in wounds treated with hydrogels co-enriched with lavender oil and ofloxacin on the 10th day when compared to the blank hydrogels [65].

Tajik et al. formulated keratin/PVP hybrid hydrogels loaded with lavender oil extract via UV irradiation for bacteria-infected wounds [66]. The physicochemical properties and successful formulation of hydrogels enriched with lavender oil were confirmed by FTIR and XRD analysis. The lavender-oil-enriched hybrid hydrogels displayed lower swelling capability when compared to the plain PVP hydrogel owing to the development of interpenetrating networks through hydrogen bonding between keratin and PVP. The in vitro drug release analysis displayed initial burst release that may be associated with the rapid release of lavender from the swollen hydrogel, followed by the plateau after 8 h that is mainly related to lavender extract diffusion and disruption of a hydrogel. The in vitro antibacterial studies of hybrid hydrogels loaded with lavender oil extract using the agar diffusion method displayed good antibacterial properties against both *E. coli* and *S. aureus*, confirming their effective applications in the treatment of bacteria-infected wounds [66].

### 5.2. Hydrogels Enriched with Tea Tree Oil

Tea tree oil (TTO) is an essential oil that is obtained from the terminal branches and leaves of the Melaleuca alternifolia [67]. Melaleuca alternifolia is a well-reputed plant in traditional and folk remedies and remains of interest in modern medicine because of its prolonged historic position as a healing agent [68]. TTO consists of a mixture of ~100 various components, mostly sesquiterpenes and monoterpenes, from which 1,8-cineole and terpinen-4-ol are the most active (antibacterial, analgesic, anti-inflammatory, antifungal, antiprotozoal, antiviral) [69,70]. Currently, the beneficial properties of the TTO and its constituents have been alternatively integrated into different products, including dermatological ointments and creams [14]. Low et al. fabricated ionically crosslinked chitosan hydrogels co-loaded with TTO and Ag^+^ ions for the treatment of bacteria-infected wounds. The in vitro antibacterial experiments using a standard well diffusion procedure showed excellent antimicrobial activity of dual drug-loaded hydrogels when incubated overnight with the following microbial strains: *S. aureus*, *P. aeruginosa*, and *C. albicans*. Combining TTO and Ag^+^ ions into the chitosan-based hydrogels further enhanced the antimicrobial efficacy by lowering the effective concentrations needed [71].

Flores et al. reported Carbopol^®^ Ultrez hydrogels enriched with TTO nanocapsules and nanoemulsions for wound management [72]. These hydrogels showed pH values that range between 5.6 and 5.8, which were close to the pH of the human skin, indicating that they cannot cause skin irritation. The spreadability analysis of hydrogels displayed high spreadability that ranges between 4.53 ± 0.22 and 9.06 ± 1.64 mm^2^/g, indicating that these hydrogels can deliver an adequate dose of the bioactive agent to the skin during wound dressing. These hydrogels significantly reduced ear oedema after the exposition of UVB radiation. A reduction of about 70% was obtained for both hydrogel-enriched TTO nanocapsules and nanoemulsions when compared to the untreated group. The in vivo wound healing experiments using cutaneous wounds in rats showed that the hydrogels enriched with TTO nanocapsules and nanoemulsions were more effective in the treatment of wounds. The histological studies revealed that hydrogels containing TTO nanocapsules and nanoemulsions resulted in faster elimination of crust compared to the pristine hydrogels, suggesting that the TTO nanostructured systems accelerated the process of re-epithelialization [72].

Altaf et al. prepared PVA/starch hydrogels containing TTO for wound dressing applications. The water vapour transmission rate (WVTR) of TTO-enriched hydrogels was 45.63 ± 2.28 g/m^2^h, indicating the ability of the hydrogels to provide moderate moisture for the acceleration of the wound healing process. The in vitro antibacterial analysis of TTO-loaded hydrogels employing the disc diffusion procedure showed good antibacterial activity against *E. coli* and multi-resistant *staphylococcus aureus* (MRSA), although it was not superior to clove-oil-loaded hydrogels. This may be due to the major component of the TTO (terpinen-4-ol) that showed resistance against both *E. coli* and MRSA [73]. The alginate-based hydrogels loaded with TTO microemulsion reported by Catanzano et al. exhibited excellent antibacterial efficacy against *E. coli*, resulting in strong inactivation of bacteria after 6 h already, while a complete disruption was accomplished after approximately 12 h [70].

### 5.3. Hydrogels Enriched with Thyme Oil

**Figure 7:**
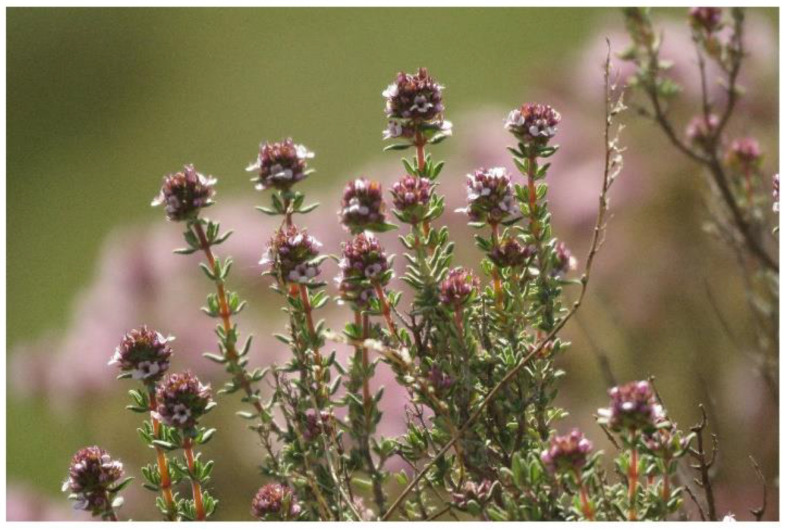
Thymus vulgaris [74].

Thyme oil is an essential that is extracted from fresh leaves and flowers of thyme (*Thymus vulgaris*) (Figure 7) [74] through steam distillation [75,76]. This essential oil is well recognized as a human medicine with antioxidant and antimicrobial properties against a broad spectrum of Gram-positive and Gram-negative bacteria strains [77]. Thyme oil consists of more than 60 active components, particularly carvacrol thymol, rosmarinic acid, and phenols thymol [78]. It hinders the growth of bacteria outside and within the body, such as bacterial infections in the urethra and genitals, respiratory system, and intestines as well as external exposure to skin wounds [79]. Therefore, thyme essential oil is a potential bioactive agent for application in wound healing. The thyme-oil-enriched cellulose hydrogels formulated by Lu et al. exhibited excellent antibacterial activity against *E. coli* and *S. aureus*, suggesting their effectiveness as wound dressing materials for the treatment of bacteria-infected wounds [80]. The in vitro drug release from the hydrogel was an initial burst release of thyme oil for the first 24 h followed by a slow and sustained drug release, revealing that the thyme-oil-loaded hydrogels possess the ability to kill fast bacteria and protect wounds from further infections [80]. 

Boccalon et al. designed sodium alginate/PVA hybrid hydrogels enriched with thyme oil [81]. The in vitro antimicrobial experiments were performed using the Kirby–Bauer disk diffusion method, and their results exhibited good antibacterial activity against both Gram-negative (*P. aeruginosa*) and Gram-positive (*S. epidermidis* and *S. aureus*) bacteria and yeast (*Candida albicans*). The plain hydrogel did not display any antimicrobial activity [59]. These antimicrobial outcomes demonstrated that the thyme-oil-enriched hybrid hydrogels are potential dressings for wound healing of bacteria-infected wounds. Koosehgol et al. fabricated chitosan/PEG hydrogels enriched with thyme oil [82]. The WVTR of the hydrogels was 1300 g·m^−2^·day^−1^ for the hydrogel loaded with a high content of thymol (1.8%(*v*/*v*)), indicating that these hydrogels can be used in moderate exuding wounds and that other blend hydrogels with lesser WVTR values are suitable for low exudate ones. The antibacterial analysis showed that the chitosan/PEG hydrogels against *E. coli* and *S. aureus* increased with increasing amounts of thyme oil [82].

The thyme-oil-enriched chitosan-based hydrogels formulated by Moradi et al. exhibited high cell migration and adhesion of the fibroblasts (L929 cells) with cell viability that ranged between 69 and 72.5% after 24 h, indicating that these hydrogels are non-toxic and possess excellent biocompatibility, both of which are properties of ideal wound dressings [83]. Rong et al. prepared chitosan/cellulose hydrogels enriched with thyme oil for application in wound dressing. The in vitro antibacterial analysis of hydrogels using the Agar disc diffusion method showed that there were not any zones of inhibition against *S. aureus* and *E. coli* in the blank hybrid hydrogels (used as control), revealing that they had no antibacterial efficacy, while the thyme-oil-enriched hydrogels produced visible zones of inhibition of the average radius of not less 2 cm in all, suggesting excellent antibacterial activity [84]. The thyme-oil-loaded gelatin/silk sericin hydrogels formulated by Chuysinuan et al. showed an initial rapid release of thyme oil in the first hour of in vitro drug release analysis, followed by a sustained release. The in vitro antimicrobial studies employing the agar diffusion method revealed that the hydrogels containing thyme essential oil inhibited bacterial growth of *S. epidermidis* and *S. aureus*, demonstrating that the antibacterial gelatin/silk sericin hydrogels enriched with thyme oil are potential wound dressings [85]. Singh et al. loaded thyme oil into hydrogel membranes prepared from a combination of κ-carrageenan and polyethylene glycol [86]. The hydrogels provided a moist environment for the wound bed to prevent infections and accelerated wound healing. The antibacterial property of hydrogel was enhanced by loading the essential oil with a 95% antimicrobial activity against Gram-positive and Gram-negative bacteria. The antibacterial activity of the hydrogel was influenced by the content of the essential oil in the hydrogel, with 15% of the essential oil in the hydrogel exerting a significant antibacterial effect. The hydrogels were non-cytotoxic on HEK293 cells, which were confirmed by 80% cell viability. The release profile of the oil from the hydrogel was slow and appropriate for long-term antibacterial effects [86]. 

### 5.4. Hydrogels Enriched with Other Essential Oils 

Other essential oils demonstrate interesting properties that can be beneficial in the treatment of wounds, including rosemary oil, *oregano oil*, *St John’s Wort* or *Hypericum perforatum* [87], *Lemongrass*, eucalyptus, *Cinnamon*, *Peppermint oil*, etc. Several pre-clinical reports have shown that polymer-based hydrogels encapsulated with these essential oils are potential scaffolds that can be used in wound treatment. Rosemary oil is an essential oil that is extracted from the aromatic herb called *Rosmarinus officinalis* L. (Lamiaceae), a woody perennial herb, found in the Mediterranean region. *R. officinalis* has been employed in folk medicine for the treatment of poor circulation, headaches, mild analgesic, anti-inflammatory, and epilepsy [88]. Rosemary essential oil exhibits excellent antimicrobial and antioxidant properties as well as wound healing efficacy [89]. Gavan et al. formulated carbopol hydrogels loaded with ethanol extract of rosemary oil and their antimicrobial results showed good antibacterial results against *S. aureus* and *P. aeruginosa*, revealing them as potential wound dressings for the management of bacteria-infected wounds [90]. 

*Eupatorium adenophorum* is a plant that belongs to the Asteraceae family, and it has been employed for a wide range of various medical treatments (blood coagulant, antimicrobials, analgesic, antiseptic, and antipyretic) for centuries, mainly in traditional Chinese medicine [91]. Chuysinuan et al. prepared gelatin-based hydrogels loaded with *Eupatorium adenophorum* essential oil for wound dressing [92]. The mechanical studies showed that the essential-oil-loaded hydrogels possessed good mechanical properties with Young’s modulus that ranges between 938.43 ± 150.39 and 1691.21 ± 248.83 MPa, and stress at maximum load that ranges from 10.16 ± 2.53 to 22.95 ± 9.76 MPa, and percentage strain at yield ranging between 1.53 ± 0.60 and 2.48 ± 0.42, revealing that they can be easily used on wounds. The in vitro antimicrobial analysis showed that *Eupatorium adenophorum*-oil-enriched hydrogels are more effective against *S. epidermidis*, *S. aureus*, *E. coli*, and *B. cereus,* while pristine hydrogels did not show any antibacterial activity [92]. Huerta et al. formulated cellulose nanofiber hydrogels enriched with clove essential oil emulsion for application in skin regeneration. The in vitro cytotoxicity studies using the alamarBlue^®^ assay showed that the hydrogels displayed high cell viability on human gingival fibroblast cells in the range of 74–100%, suggesting excellent biocompatibility and non-toxicity of clove-oil-loaded hydrogels, an interesting feature for ideal wound dressings [93]. 

Some studies reported interesting biological activities that are exhibited by ginger essential oil, including antibacterial, anti-inflammatory, and antioxidant activities, which are very important in the treatment of chronic or infected wounds [94,95]. Ngampunwetchakul et al. formulated Semi-solid PVA-based hydrogels enriched with ginger essential oil loaded in chitosan nanoparticles for application in wound treatment. The indirect cytotoxicity studies of the systems showed cell viability that ranges from 83–103% to 81–95% for the Normal Human Dermal Fibroblasts cell line (NHDF) and NCTC clone 929 cells, respectively, indicating their excellent biocompatibility and non-toxicity [96]. The ginger extract-enriched gelatin/PVA hydrogels formulated by Khan et al. The hydrogels exhibited similar in vivo wound-healing efficacy on burn wounds on the backside of the rabbits when compared to the commercial wound dressing, while much wound-healing activity was observed as compared to the control group confirmed by the intensive formation of collagen in the histopathological analysis [97].

Gherman et al. designed gelatin-based hydrogels loaded with *Galium verum* essential oil for bacterially infected wounds. The in vitro antibacterial analysis evaluated by the agar diffusion procedure exhibited excellent antibacterial activity against *S. aureus* and *E. coli* with a ZOI of 17–26 and 18–28, respectively [98]. *Zataria multiflora* is a thyme-like plant belonging to the *Lamiaceae* family that originates from Iran and is broadly employed as a flavouring ingredient in a broad variety of fields in its native region, The biological properties of *Zataria multiflora* include antioxidative and antimicrobial properties [98]. Kavoosi et al. gelatin–PVA hybrid hydrogels are enriched *with Zataria multiflora* essential oil for wound treatment. The antibacterial experiments of *Zataria multiflora* essential-oil-enriched hydrogels showed excellent antibacterial efficacy against all bacteria strains (*E. coli*, *S. aureus*, and *Bacillus cereus*) except *P. aeruginosa*, which was resistant. These results revealed that hydrogels loaded with essential oils are potential candidates that can be used in the treatment of bacteria-infected wounds [99]. Wang et al. loaded selected essential oils into hydrogels, such as eucalyptus, ginger, and cumin oil. The hydrogels were prepared by physical crosslinking of carboxymethyl chitosan and carbomer 940. The hydrogels loaded with eucalyptus oil displayed optimal antibacterial activities against *S. aureus* and *E. coli* (46.26  ±  2.22% and 63.05  ± 0.99%, respectively) with cell viability of over 92.37%. The hydrogel also enhanced wound healing in mouse burn models in vivo with a significant formation of dermis and epidermis. The wound repairs were characterized by downregulation of interleukin-6 and tumour necrosis factor-α with an upregulation of the transforming growth factor-β, vascular endothelial growth factor and epidermal growth factor [100]. The hydrogel revealed the potential to repair skin burn wounds. The rough surface of the hydrogel is also a good feature for cell attachment and wound healing. 

*Cannabis sativa* L. (hemp), a plant of the *Cannabaceae* family, contains cannabinoids, which have been reported to be effective for treating pain and inflammation associated with wounds [101]. Wang et al. reported a combination of *Cannabis sativa* oil with silver nanoparticles loaded into collagen hydrogels. The hydrogel inhibited 99% of *S. aureus* and *P. aeruginosa* [102]. The mode of action of the main constituent in the oil is via inhibition of the peptidoglycan, RNA, synthesis of proteins, DNA, and the disruption of cytoplasmic membranes in the bacteria. Combining the essential oil with nanoparticles resulted in synergistic antibacterial effects [103]. A summary of hydrogels loaded with essential oil for wound dressing is shown in Table 1. There are some commercially available wound dressings loaded with essential oils (Table 2).

## 6. Conclusions and Future Perspective

Microbial invasion of the wound bed results in infections and is the main factor that contributes to chronic wounds. The treatment of chronic wounds using antibiotics is hampered by drug resistance. Furthermore, some of the commercially available wound dressing products also suffer from the poor antimicrobial activity and do not protect the wound from bacterial invasion. Hydrogels have attracted much attention in the biomedical field due to their promising features. However, some prepared hydrogels exhibit poor antibacterial effects. The loading of essential oils into hydrogels impart interesting features, making them ideal for wound dressing, such as moderate WVTR, excellent biocompatibility, non-cytotoxicity, soothing effects, and good antimicrobial properties. The essential oil-enriched hydrogels display good antibacterial effects against many clinical bacteria strains that can cause wound infections. However, most of the essential oil loaded into the hydrogel displayed selective antibacterial activity against some strains of bacteria. The concentration of essential oils loaded into the hydrogels also influenced their antibacterial effects. There are currently few pre-clinical studies on essential-oil-enriched hydrogels and there is a pressing need to fully understand their mode of action in wound healing. Wound healing studies using animal models for hydrogels loaded with essential oils are few. Further studies are also lacking in current literature, such as the stability of the loaded essential oils in wound dressings, in vivo studies, potential toxic effects, and their antibacterial mode of action. There is no doubt that more studies on hydrogels loaded with essential oils will translate to clinical use. 

## Figures and Tables

**Table 1:** A summary of the therapeutic efficacy of polymer-based hydrogels enriched with essential oils.

Polymers Used	Loaded Essential Oils	Outcomes	References
Gellan gum	Lavender oil	Excellent antibacterial and antioxidant activity, and fast wound healing process.	[65]
Keratin and PVP	Lavender oil	Good antibacterial properties	[66]
Chitosan	TTO	Excellent antimicrobial activity.	[67]
Carbopol	TTO	Excellent spreadability and effective wound healing efficacy.	[72]
PVA and starch	TTO	Moderate WVTR and good antibacterial activity.	[73]
Alginate	TTO	Excellent antibacterial efficacy	[70]
Cellulose	Thyme oil	Burst drug release followed by sustained release. Excellent antibacterial activity.	[80]
Alginate and PVA	Thyme oil	Excellent antimicrobial activity.	[82]
Chitosan and PEG	Thyme oil	Moderate WVTR and good antibacterial efficacy	[82]
Chitosan	Thyme oil	Excellent biocompatibility and non-toxicity.	[83]
Chitosan and cellulose	Thyme oil	Excellent antibacterial activity.	[84]
Gelatin and silk sericin	Thyme oil	Initial rapid drug release followed by sustained release. Excellent antibacterial activity.	[85]
κ-carrageenan and polyethylene glycol	Thyme oil	The hydrogels prevented infections and accelerated wound healing with 95% antimicrobial activity against Gram-positive and Gram-negative bacteria and non-cytotoxic.	[86]
Carbopol	Rosemary oil	Good antibacterial activity.	[90]
Gelatin	*Eupatorium adenophorum* oil	Good mechanical properties and antibacterial activity.	[92]
Cellulose	Clove oil	Excellent biocompatibility and non-toxicity.	[93]
PVA	Ginger essential oil	Excellent biocompatibility and non-toxicity.	[96]
Gelatin and PVA	Ginger oil	Good wound healing activity.	[97]
Gelatin	*Galium verum* essential oil	Excellent antibacterial activity.	[98]
Gelatin and PVA	*Zataria multiflora* essential oil	Excellent antibacterial activity.	[99]
carboxymethyl chitosan and carbomer 940.	Eucalyptus, ginger, and cumin oil.	Excellent antibacterial activities against *S. aureus* and *E. coli* (46.26 ± 2.22% and 63.05 ± 0.99%, respectively) with cell viability of 92.37%.	[100]
Collagen	Cannabis sativa oil	The hydrogel inhibited 99% of *S. aureus* and *P. aeruginosa*.	[102]

**Table 2:** Commercially available wound dressings loaded with essential oils.

Wound Dressings	Type of Wound Dressings	Essential Oils	Therapeutic Outcomes	References
Camisan^®^	Topical cream	Chamomile oil	Accelerated healing, granulation, and epithelialization. Effective for leg varicose ulcers and decubitus ulcers.	[103]
Hyperoil^®^	oil, gel, cream and gauze gel.	Neem oil	Prevent infection, supports re-epithelialization and offers a soothing effect. Effective for diabetic foot ulcers.	[104]

## Data Availability

The data presented in this study are available on request from the corresponding author.
